# Towards a risk-based food safety management system in the fresh produce supply chain in Da Nang, Viet Nam

**DOI:** 10.1016/j.heliyon.2024.e32701

**Published:** 2024-06-11

**Authors:** Thanh Ha Thi Mac, Thi Dong Phuong Nguyen, Minh Nhat Dang, Thi To Quyen Ta, Pauline Spagnoli, Mieke Uyttendaele, Liesbeth Jacxsens

**Affiliations:** a Department of Food Technology, Faculty of Chemical Engineering, University of Science and Technology, The University of Da Nang, Da Nang City, 550000, Viet Nam; b Department of Food Technology, Safety and Health, Faculty of Bioscience Engineering, Ghent University, Coupure Links 653, 9000, Ghent, Belgium

**Keywords:** Food safety management, Viet Nam, Fresh produce, Microbiological hazards, Good agricultural practices

## Abstract

Food safety has emerged as a paramount concern for both Vietnamese consumers and the government. However, limited data are available on food safety management systems in Viet Nam. This study identified significant gaps in good agricultural and hygienic practices along the fresh produce chain (farmers and traditional wholesalers/market sellers) in the region of Da Nang, Viet Nam. This was achieved through a survey on good agricultural and hygienic practices for farmers (n = 100) and sellers (n = 100), which researchers further supplemented by microbiological analysis for *E. coli, Salmonella* spp., and *Listeria monocytogenes* on leafy greens, water in contact with produce and contact surfaces (hands). The results indicated that 86.0 % of farmers and 54.0 % of sellers received food safety training in the last 3 years; and women dominated both vegetable cultivation but also trading. Farm-level deficiencies included inadequate handwashing practices, lack of documentation for manure application schedules, improper washing and drying of harvest tools, failure to keep containers elevated off the ground, improper storage of vegetables, and inadequate covering of containers, with respectively 34.0 %, 30.3 %, 12.1 %, 41.7 % and 7.9 % of farmers executing the practice as prescribed by the WHO/FAO ‘5 keys of growing safer fruits and vegetables’. As for sellers, the most dominant gaps (<50.0 % compliance) were the way of handwashing and the practice of keeping containers elevated off the ground before, during, and after harvesting. The microbiological analysis confirmed that, in a total of 36 fresh produce samples including mustard greens, cucumber, lettuce, and crown daisy, the number of samples positive for *E. coli*, *Salmonella* spp., and *L. monocytogenes* were 12, 2, and 10 respectively. Samples of hands and the irrigation water showed high contamination with *E. coli*. Based on identified gaps, risk communication tools were developed and distributed amongst farmers, sellers, and Da Nang food safety management authority (governmental organisation performing inspections in the traditional food markets). As intervention, two farmers and two sellers were trained in safe agricultural practices for the cultivation of fresh vegetables (managerial intervention) and instructed to use tap water as irrigation water instead of uncontrolled surface water (technological intervention). A post-assessment was conducted, including redoing the survey on good practices and microbiological analysis. The outcome of these interventions showed positive results in terms of good agricultural and hygienic practices resulting in improved hygiene levels and safety of the fresh produce. The findings from this research have the potential to provide a model for the development of a science-based risk management strategy in alternative food chains or geographic areas in emerging countries.

## Graphical abstract

**Figure:**
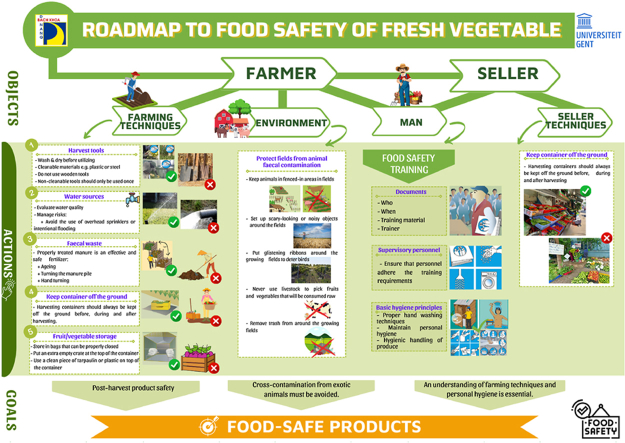

## 1 Introduction

In Viet Nam, but also in other Asian countries, the agri-food chain often consists of smallholders or small to medium companies with limited vertical integration, and hence limited uniformity and control in the supply chain is achieved. Food safety has become one of the top concerns of Vietnamese consumers and the government. The report from the National Assembly on monitoring the implementation of policies and laws on food safety showed that from 2011 to 2016, there were 668673 foodborne disease cases, and on average twenty-one deaths per year in Viet Nam [1]. In addition, during the same period, 1007 food poisoning outbreaks with 30395 food poisoning cases had been reported [1]. Data and statistics have shown that in the first quarter of 2022, there were four cases of food poisoning, resulting in ninety-one people poisoned and thirty-five people died [2].

Ready-to-eat fresh produce is defined as ‘fresh fruits and vegetables to be eaten raw, without peeling, cooking or other handlings to reduce microbiological contamination’ (Commission Notice (2017/C 163/01), 2017). As no reduction of potential microbiological contamination with pathogens such as *Listeria monocytogenes* or *Salmonella* spp. occurs before consumption, preventive measures along the food supply chain are needed to reduce the risk of contamination from farm to plate. This can be achieved by the implementation of a food safety management system, based on good agricultural and hygienic practices in each actor of the supply chain. This approach has globally been widely demonstrated to be effective (e.g. [3] (global data on leafy greens)) but also in the context of emerging countries (e.g.[4] (basil production of South Africa) [5]; (lettuce production in South Brazil) [6]; (lettuce production of South Africa)). Major risk factors are contaminated (often uncontrolled, untreated) surface water, overhead irrigation systems, lack of personal hygiene (hand washing, toilets), intrusion of (wild) animals, (under)composted manure and use of unclean harvest/post-harvest tools and recipients [7]. These situations can introduce fecal contamination and associated fecal pathogens as *Salmonella* spp. or pathogenic *E. coli.* The challenges encountered by agri-food systems, such as maintaining accurate farm management, product marketing, and input access can also be resolved with the help of information and communication technologies as demonstrated by Hasan et al. [8] who employed an autoregressive distributed lag (ARDL) model to determine the long- and short-run dynamics of variables related to ICT and sustainable agriculture in Bangladesh. However, it would not be feasible for farmers in remote locations to have internet access and mobile networks to consistent connectivity or for untrained farmers to locate useful data, make good use of agricultural apps, and efficiently apply digital tools.

The emergence of new and more stringent food safety standards in most industrialized countries is the result of several factors including the growth in trade of perishable high value products (such as fresh produce), advances in methods for detection of hazards and epidemiological approaches, scientific and regulatory consensus on best approaches to risk management and the recognition of global standards and approaches under the WTO [9]. This puts pressure on the local fresh produce supply chain and competent authorities of low and middle-income countries to act accordingly, as has been demonstrated by Kirezieva et al. [10] (Europe versus global context) and Nanyanja et al. [11] (context of Uganda, Kenya versus Europe). Vegetables certified as safe represent less than 10 % of the total sold in Viet Nam, with little evidence that certified products are actually safer than traditionally produced and marketed vegetables [12]. In 2018, the government launched VietGAP Vietnamese Good Agricultural Practices, 2022, a third-party certification based on GlobalGAP, as the main global standard and guidelines for safe fruit and vegetable production. Its high cost, complexity and extensive paperwork make it largely inaccessible to the majority of farmers. As a result, while the total area of vegetable production in Viet Nam is estimated to be about 735 000 ha, about 63 000 ha (8 %) have a VietGAP certificate [13]. In the Da Nang region, safe vegetables account for 6.5 % of all the vegetables supplied in the city but 75.0 % of it is sold as conventional vegetables by wholesalers in terms of price and packaging. At the retail level, 0.25 % of vegetables consumed in Da Nang are proved to be safe [14]. According to Da Nang's Department of Agriculture and Rural Development, the city consumes about 140 000 tons of vegetables and fruits every year. Of this, about 9000 tons (6.5 %) are produced in Da Nang and 131 000 tons (93.5 %) are delivered from other provinces (i.e. Gia Lai (40.0 %) and Lam Dong provinces (30.0 %)). Although modern markets are increasingly selling traceable food with proper quality assurance, the city is still struggling to ensure that reliable safe food is supplied to traditional markets, the main shopping place for most. Providing a population with healthy diets from sustainable food systems is an immediate challenge and vegetables are an important part of such a healthy diet [15]. In 2015, Da Nang city adopted a “Masterplan for safe vegetables” that aims to triple the area dedicated to safe vegetable production by 2020. Since December 2016, Da Nang has signed multiple partnership agreements with external provinces to develop safe food chains to supply the city. The authorities have also actively developed safe food supply chains with a series of major private sector actors focusing on consumers' access to certified products, sample testing, government monitoring and traceability.

There is limited research on food safety management and few data available on microbiological status in the food supply chain in Vietnam in general, and in Da Nang in particular. This makes it difficult to develop evidence-based policies and strategies to sustainably promote further development of food safety management system in Da Nang, Vietnam. Therefore, the lack of research focused on the context of Viet Nam is a significant issue that needs to be addressed.

In this research, the main risk factors on microbiological contamination in Da Nang's fresh vegetable value chain were identified based on an evidence-based analysis of food safety gaps. This was achieved through data collection on the state of the art of fresh produce chain (microbiological analysis) and current agricultural and hygienic practices (questionnaires). Based on the gap analysis, factsheets and a roadmap for improvement were designed and spread as training and risk communication tools for the farmers and sellers active in the traditional markets. Furthermore, a small pilot study was conducted with these developed materials as a training intervention with two selected farmers and two selected sellers. The impact of these interventions on good agricultural and hygienic practices and the microbiological hygiene and safety of ready-to-eat fresh produce was evaluated. This research zooms in on the specific situation of the fresh produce chain in Viet Nam, disclosing novel data on food safety practices and hygiene levels. The study discusses how scientific evidence can foster a risk-based food safety management strategy, in particular in an agri-food chain consisting of small to medium companies with limited vertical integration, to enhance food safety in a sustainable, local food system. The paper can serve as a model for science-based risk management in other food chains and/or in other regions in emerging economies.

## 2 Materials and methods

### 2.1 Study area within Da Nang's vegetable chain

**Fig. 1:**
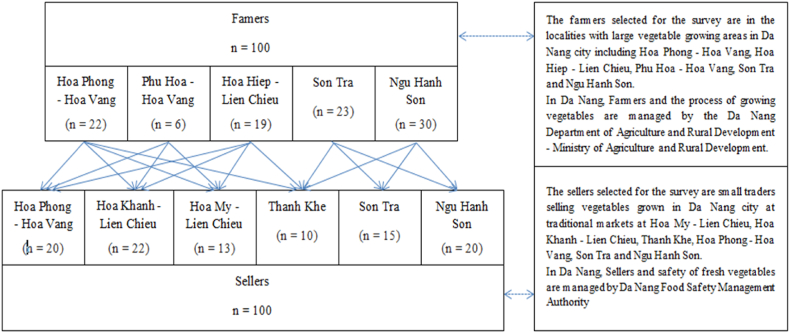
Identification and technical explanation of the study area along the Da Nang vegetable chain.

The focus of the research was purposefully put on local food supply and traditional markets, not on more established long distance vegetable supply chains delivering to supermarkets, as “VietGAP” is already implemented in those cases to ensure good agricultural and hygienic practices [16]. Fig. 1 displays the identification and technical explanation of the study area. A total of one hundred farmers in five different locations participated in the survey of good agricultural and hygienic practices, and three of those farmers participated in the microbiological analysis. The Da Nang Department of Agriculture and Rural Development, belonging to the Ministry of Agriculture and Rural Development, is the policy making body for these farmers. In addition to the farmers, small local sellers were selected from different traditional markets in Da Nang city. A total of one hundred sellers, spread across six local food markets, joined the research. The safety and hygiene of the fresh produce in the food markets is the responsibility of the Da Nang Food Safety Management Authority.

### 2.2 Questionnaire on good agricultural and hygienic practices

#### 2.2.1 Development of survey on good agricultural and hygienic practices

The first step in setting up the questionnaire on agricultural and hygienic practices included an analysis of current prescribed good agricultural and hygienic practices (e.g., application of treated manure, quality of irrigation water, infrastructure, recipients to collect produce, hygienic handling of operators) along the fresh produce chain. After scanning the research field, a diagnostic tool to measure the maturity of food safety management systems in the horticultural food chain [17]; Nanyunga et al., 2015 applied in Brazil Rodigues et al., 2014 and Rwanda Kamana et al., 2017 and the ‘5 keys of growing safer fruits and vegetables’ [7] were used to develop a first draft of the questionnaire for the farmers and sellers, translated in Vietnamese and pilot tested (to test understandability) with a limited number of farmers and sellers. Minor adaptations were made. The full questionnaire is available in Appendix 1 (farmers) and Appendix 2 (sellers).

#### 2.2.2 Administration of the survey on good agricultural and hygienic practices

To gain information on the variability of the current practices in the chain, one hundred farmers and one hundred sellers were interviewed face-to-face in the time period from January 2021 to March 2021. The sample was a convenience sample and not fully representative for the Da Nang region due to voluntary participation. The farmers selected for the survey were in localities with large vegetable growing areas in the Da Nang region. The sellers selected for the survey were small traders selling vegetables grown in Da Nang city at traditional markets (Fig. 1). The survey was filled in on paper by trained food technology bachelor students (Faculty of Chemical Engineering, University of Science and Technology, Da Nang), during a face-to-face interview conducted with each farmer and each seller. Farmers and sellers were continuously treated anonymously in further data processing.

#### 2.2.3 Data processing and gap analysis

Obtained results on current practices were analyzed in the gap analysis to identify gaps in compliance (as e.g., described in the work of [18]). This was done by calculating percentages of compliance of the farmers/sellers to the prescribed good agricultural and hygienic practices (e.g. application of composted manure). Color codes were assigned to each practice based on the level of compliance: when less than 50.0 % of farmers/sellers executed the practice as prescribed by the ‘5 Keys of growing safer fruits and vegetables’ [7], color code red was given and this practice was considered a gap, when between 50.0 % and 75.0 % of farmers/sellers complied color code yellow was given and when more than 75.0 % of farmers/sellers complied color code green was assigned (based on [19,20]). Statistical analyses were done to assess if various levels in compliance could be identified between different farms, types of farms and based on farming activities. For the sellers, comparisons were made based on educational background, experience, and training. Chi-Square Tests of Independence, with the statistical significance level established at 5 %, were applied in IBM SPSS Statistics version 28 (Chicago, Illinois).

### 2.3 Microbiological sampling plan and analysis

A microbiological sampling plan along the fresh produce chain was developed based on an assessment scheme as exemplified by Uyttendaele et al. [21], Ceuppens et al. [3], Rodrigues et al. [5]. *Salmonella* spp. and *Listeria monocytogenes* were identified as major pathogens, and *E. coli* as a generic indicator for fecal hygiene on produce, in water and on hands of the workers. Table 1 shows the assessment scheme identifying the critical sampling locations (CSL), number and type of samples, microbiological parameters, and interpretation of results. The microbiological sampling and analysis were restricted to three farms and two sellers.

**Table 1:** Descriptions of critical sampling locations (CSL), number of samples, microbiological parameters, analytical methodology, and interpretation of results.

Place in chain	CSL	Description - sampling location	Samples (n)	Microbiological parameters	Methodology	Interpretation of the results	References
Farms	1	Harvested vegetables ready to leave farm - Mustard greens - Cucumber - Lettuce - Crown daisy	24	*E. coli* *Salmonella* spp. *Listeria monocytogenes*	ISO 16649–2 (1999) ISO 6579 (2002) ISO 11290–2 (1998/Amd 1/2004)	2–3 log_10_ CFU/g Absent in 25 g Absent in 25 g	EU Regulation 2073/2005 [22] [23]
	2	Irrigation water at source + indication of source (bore hole, irrigation canal, river, lake)	3	*E. coli* enumeration	ISO 16649–2 (1999)	2 log_10_ CFU/100 ml	Commission Notice, 2017
	3	Swabs of hand of workers	5	*E. coli* enumeration	ISO 16649–2 (1999)	≤0.7 log CFU/25 cm^2^ (below limit of detection)	Jacxsens et al. [24]
Markets	4	Vegetables being sold: - Mustard greens - Cucumber - lettuce	12	*E. coli* *Salmonella* spp. *Listeria monocytogenes*	ISO 16649 (1999) ISO 6579 (2002) ISO 11290–2 (1998/Amd 1/2004)	2–3 log_10_ CFU/g Absent in 25 g Absent in 25 g	EU Regulation 2073/2005 [22] [22]
	5	Rinse water/wash water (at source, no contact with vegetables)	2	*E. coli* enumeration	ISO 16649–2 (1999)	2 log_10_ CFU/100 ml	Commission Notice, 2017
	6	Swabs of hand/glove	2	*E. coli* enumeration	ISO 16649–2 (1999)	≤0.7 log CFU/25 cm^2^ (below detection limit)	[24]

#### 2.3.1 Sampling locations

At farm level, samples were taken from the fresh produce, i.e. harvested vegetables that were ready to leave the farm (CSL 1). Vegetables included a fruit vegetable (cucumber) and leafy green vegetables (i.e. lettuce, mustard greens, and crown daisy). Water samples were taken from the irrigation water source of the farm (CSL 2) and swabs from the hands of workers on the farm (CSL 3) were collected. In the traditional markets, samples included vegetables (i.e. lettuce, mustard greens, and cucumber) - (CSL 4), water used for washing the vegetables (CSL 5), and swabs of the hands or gloves of sellers - (CSL 6) (Table 1).

#### 2.3.2 Sampling method

Thirty-six randomly selected samples of fresh vegetables (ten mustard greens, nine cucumbers, ten lettuces, and seven samples of crown daisy) were collected with gloves and put directly into (sterile) clean plastic bags. Five samples of water, approximately 100 ml each, were collected into sterilized bottles. For the sampling of the hand or glove of the workers, a swab was removed from the sterile wrapping, after which the tip was moistened by immersing it in 0.1 % peptone water (v/v). The entire hand or glove was streaked, whilst rotating the swab between thumb and forefinger in two directions at right angles [25]. The swab was placed in a tube containing 5 ml of 0.1 % peptone water (v/v) [25], cooled and transported at < 8 °C to the lab for microbial analysis within 24 h. Traceability was guaranteed through a sample coding and identification form.

#### 2.3.3 Microbiological parameters and methods of analysis

Table 1 describes the microbiological parameters and microbial analyses of samples. For the *E. coli* identification, HiCrome TM *E. coli* Agar (Himedia) was prepared in petri plates. Twenty-five grams of the samples were mixed with 225 ml of BPW (Buffered peptone water) and were homogenized in a stomacher for 30 s. Then, a serial dilution was prepared, and 1 ml of each sample was were spread on HiCrome TM *E. coli* Agar plates (spread plate). Each dilution was repeated three times. The plates were incubated for 4 h at 30 °C, and then for 18 h at 44 °C. The confirmation colony was shown in bluish green (ISO 16649–2:1999) [26].

To detect *Salmonella* spp., the horizontal method described in ISO 6579:2002 was used [27]. First, 25 g of the samples were mixed with 225 ml of BPW (Buffered peptone water) and incubated at 37 °C for 18 h (±2 h). Then, 0.1 ml of the samples was mixed with 10 ml RVS broth (Titan), incubated at 41.5 °C ± 0.5 °C for 24 h ± 3 h. In parallel, 1 ml of the samples was mixed with 10 ml MKTTn broth (Titan) and was incubated at 37 °C ± 1 °C for 24 h ± 3 h. After the enrichment procedure, the inoculum was plated onto two selective isolation media, XLD agar (Himedia) and RAPID’*Salmonella* agar (Himedia) and incubated at 37 ± 1 °C for 24 h ± 3 h. After incubation, the XLD and RAPID’*Salmonella* plates were examined for the presence of typical *Salmonella* spp. colonies. Typical colonies of *Salmonella* spp. usually have a black center with transparent white or red border on XLD agar and are pink to purple (magenta) on RAPID’*Salmonella* agar. Five colonies from either medium (or spread over both) were selected and transferred with a loop onto TSA agar plates, incubated at 37 °C ± 1 °C for 24 h ± 3 h. The pure cultures on TSA agar were used for the biochemical confirmations (*Salmonella* identification kit, Himedia).

*Listeria monocytogenes* was identified according to ISO 11290–2 (1998/Amd 1/2004) [28]. Twenty-five grams of the sample was mixed with 225 ml of BPW (Buffered peptone water), and homogenized in a stomacher for 30 s. Next, a serial dilution was made. 0,1 ml of each dilution was inoculated onto ALOA-plates (Himedia) and was incubated for 48 h ±3 h at 37 °C ± 1 °C in a plastic bag. The to be identified colonies on the ALOA-plates were green blue colored and surrounded by an opaque halo. These were counted and five characteristic colonies were selected for confirmation. These five colonies were transferred onto TSA agar-plates, incubated for 24 h at 37 °C ± 1 °C. The pure culture was then used for further confirmation tests such as catalase test, motility test, biochemical test (Hi *Listeria* TM identification kit, Himedia).

#### 2.3.4 Microbiological guidance values applied for interpretation of results

As no legal requirements are yet set on fresh produce, agricultural water, or hands of workers in the food chain in Viet Nam (and many other countries), microbiological guidance values as available in Uyttendaele et al. [22] were applied (Table 1). *Salmonella* spp. and *Listeria monocytogenes*, due to their low infectious dose, should be absence in 25 g or ml water or cm^2^ hand surface. *E. coli* is applied as an indicator for fecal hygiene, and can be accepted between 2 and 3 log_10_ CFU/g produce (process hygiene criterium for fresh-cut produce according to EU Regulation 2073/2005). Lastly, it is recommended to apply high quality water (max. 100 CFU E*. coli*/100 ml) for this type of cultivation, i.e. ready-to-eat leafy greens consumed raw Commission Notice, 2017.

### 2.4 Development and dissemination of factsheets and roadmaps as risk communication material

To address the identified gaps in good agricultural practices, a factsheet was developed for each identified gap (ten factsheets in total) based on WHO/FAO's ‘five keys of growing safer fruits and vegetables’. Each factsheet was tailored to the Vietnamese situation. The factsheets were presented to and discussed with the hundred included farmers and the hundred included sellers during a feedback workshop, organized with the Da Nang Food safety authority (period March–April 2022). The updated factsheets, so those after discussion with the farmers and sellers, were applied as training material for the farmers and sellers during the supervised training intervention (see section 2.5). The updated factsheets were also applied to develop a comprehensive roadmap to facilitate access to food safety information and training as a risk comprehension tool, encouraging farmers and chain actors in daily application of good practices and food safety management. The roadmap to food safety of fresh vegetables sets out the objectives, key action areas and solutions urgently required to improve food safety management systems. The roadmap was then discussed with Da Nang Food safety authority in order to fine-tune it and tailor it to the local context. The roadmap is further applied by the Da Nang Food Safety Authority as a risk communication tool to farmers and in the traditional markets.

### 2.5 Completion of the interventions and post-assessment

Four of the study participants (two farmers and two sellers) were ready to participate in the continuation of the research. These were selected based on the availability of the farmers and the sellers to join these further steps in the research. Due to the season, mustard greens and crown daisy were chosen as training topic in the farms while lettuce, mustard greens and cucumber were selected for the traditional market.

Farmers participated in a one-day training session on good agricultural practices and received instructional materials for reference. Subsequently, a technical assistant was assigned to visit their farms three times per week during the cropping season to provide direct guidance and to monitor the two farmers on good practices and the use of municipal water as a source of irrigation water during the cultivation (technological intervention)-. After six weeks, vegetables were harvested, and the same set of analyses as displayed in Table 1 were conducted for product samples, hands, and municipal water for the post assessment.

For the sellers, managerial gaps linked to hygienic practices were identified (section 3.2.2.), namely hand washing and keeping containers off the ground. Therefore, the training was done based on these two gaps, and the sellers were followed by the researcher for four weeks. Subsequently, a post intervention sampling was also conducted with these sellers to re-evaluate the agricultural and hygienic practices (Appendix 1, Appendix 2) (Table 1). To provide a clearer explanation of the research methodology, the flowchart of current research design was depicted in Appendix 3.

## 3 Results and discussion

### 3.1 Characterization of respondents

One hundred growers of vegetables (60.0 % female and 40.0 % male, Table 2), working in different farming locations (Fig. 1), participated in this study. Farms were labeled and located in Viet Nam as follows: F1 (located at Hoa Phong ward, Hoa Vang district), F2 (located at Hoa Hiep ward, Lien Chieu district), F3 (located at Hoa Phu ward, Hoa Vang district), F4 (located in different regions in Son Tra district), F5 (located in different regions in Ngu Hanh Son district). Characteristics of respondents in these farms, like age, gender, educational background, farming experience, type of farm, type of vegetables grown, farming activities, food safety training and time passed since last training were summarized in Table 2. The results of the questionnaire show that 53.0 % of farmers had received sufficient education (high school and higher education), and that most of the farmers (86.0 %) were between 30 and 65 years old. Furthermore, most of the respondents had more than 5 years of farming experience. 59.0 % of the surveyed farmers produced leafy greens and 86.0 % of surveyed farmers received food safety training in the last 3 years. 63.0 % of farmers ran their businesses together with family members, while 37.0 % of them were members of vegetable farmers’ cooperatives or associations. Data of farming activities demonstrated a low risk of cross-contamination between plant and animal-based products (Table 2).

**Table 2:** Characterization of respondents at farm level (n = 100), expressed in percentage (%).

Parameters	Description	Overall (all farms) n = 100	Region of farms in Da Nang area				
			Hoa Phong - Hoa Vang (F1) n = 22	Hoa Hiep - Lien Chieu (F2) n = 19	Hoa Phu - Hoa Vang (F3) n = 6	Son Tra (F4) n = 23	Ngu Hanh Son (F5) n = 30
Age	15–18 years	1.0	0.0	5.3	0.0	0.0	0.0
	19–29 years	2.0	0.0	5.3	0.0	4.3	0
	30–65 years	86.0	81.8	57.9	100.0	91.3	100.0
	>70 years	11.0	18.2	31.6	0.0	4.3	0.0
Gender	Male	40.0	22.7	26.3	33.3	52.2	53.3
	Female	60.0	77.3	73.7	66.7	47.8	46.7
Educational background	None	9.0	9.1	10.5	0.0	4.3	13.3
	Elementary school	38.0	50.0	36.8	16.7	26.1	43.3
	High school	50.0	40.9	36.8	83.3	69.6	43.3
	Higher education	3.0	0.0	15.8	0.0	0.0	0.0
Farming experience	<5 years	3.0	0.0	5.3	0.0	8.7	0.0
	5–10 years	41.0	22.7	21.1	16.7	34.8	76.7
	11–20 years	31.0	59.1	36.8	50.0	34.8	0.0
	21–40 years	21.0	18.2	15.8	33.3	21.7	23.3
	>40 years	4.0	0.0	21.1	0.0	0.0	0.0
Type of vegetables grown	Leafy greens	58.0	45.5	57.9	100.0	56.5	60.0
	Fruity vegetable	33.0	36.4	31.6	0.0	34.8	36.7
	Root vegetable	3.0	9.1	0.0	0.0	4.3	0.0
	Edible plant stem	6.0	9.1	10.5	0.0	4.3	3.3
Food safety training	Yes	86.0	95.5	84.2	100.0	65.2	93.3
	No	14.0	4.5	15.8	0.0	34.8	6.7
Time passed since last training	<1 year	36.0	86.4	31.6	33.3	26.1	10.0
	1–3 years	41.0	18.2	31.6	50.0	30.4	70.0
	>3 years	23.0	18.2	26.3	50.0	17.4	23.3
Type of farm	Family business	63.0	0.0	63.2	100.0	100.0	73.3
	Cooperation	37.0	100.0	36.8	0.0	0.0	26.7
Farming activities	Solely vegetables/fruits/cereals/crops	77.0	77.3	89.5	100.0	73.9	66.7
	Livestock and crops	23.0	22.7	10.5	0.0	26.1	33.3
	Crops and aquaculture	0.0	0.0	0.0	0.0	0.0	0.0

One hundred sellers were also selected (randomly) in the regions of S1 (located at Hoa My ward, Lien Chieu district), S2 (located at Hoa Khanh ward, Lien Chieu district), S3 (located in different regions in Thanh Khe district), S4 (located at Hoa Phong ward, Hoa Vang district), S5 (located in different regions in Son Tra district) and S6 (located in different regions in Ngu Hanh Son district) (Fig. 1). From Table 3 it can be concluded that women (78.0 %) dominated the fresh produce trade in the study area. When this is compared to the farmers, where 60.0 % of respondents were women, a trend becomes apparent. Indeed, the feminization of agriculture is occurring in many countries, e.g. China [29]. In addition, women are increasingly becoming more engaged in farm management ([30], context of Kenya). Previous studies have shown that there are gender-specific domains in rural farming systems, with men taking up tasks like land preparation, while planting, fertilizing, harvesting, and cleaning are typically women's responsibility. Therefore, this could be taken into account when developing projects or policy, including gender tailored guidelines [31]. Next to gender, the other characteristics show that the majority of the sellers (67.0 %) have had formal education (high school and higher education) and 90.0 % of them were in the age category of 30–65 years old. This is a similar trend as is visible in the characteristics of the included farmers (Table 2). Furthermore, most traditional sellers (78.0 %) had more than 5 year experience, (and leafy greens are the vegetables sold most often (77.0 % of farmers sell leafy greens). 54.0 % of seller respondents had received food safety training, most probably because the turnover of the sellers is higher compared to the farmers. Farmers also received training by the Ministry of Agriculture.

**Table 3:** Characterization of respondents at seller level (n = 100), expressed in percentage (%).

Parameters	Description	Percent (%) n = 100	Region of markets in Da Nang area					
			Hoa My - Lien Chieu (S1) n = 13	Hoa Khanh - Lien Chieu (S2) n = 22	Thanh Khe (S3) n = 10	Hoa Phong - Hoa Vang (S4) n = 20	Son Tra (S5) n = 15	Ngu Hanh Son (S6) n = 20
Age	15–18 years	0.0	0.0	0.0	0.0	0.0	0.0	0.0
	19–29 years	6.0	7.7	4.5	0.0	0.0	20.0	7.7
	30–65 years	90.0	92.3	86.4	90.0	95.0	80.0	92.3
	>70 years	4.0	0.0	9.1	10.0	5.0	0.0	0.0
Gender	Male	22.0	7.7	4.5	0.0	10.0	40.0	7.7
	Female	78.0	92.3	95.5	100.0	90.0	60.0	92.3
Educational background	None	2.0	0.0	0.0	0.0	10.0	0.0	0.0
	Elementary school	31.0	15.4	27.3	30.0	35.0	33.3	15.4
	High school	65.0	76.9	68.2	70.0	55.0	66.7	76.9
	Higher education	2.0	7.7	4.5	0.0	0.0	0.0	7.7
Selling experience	<5 years	22.0	23.1	45.5	30.0	30.0	0.0	23.1
	5–10 years	46.0	15.4	31.8	70.0	40.0	66.7	15.4
	11–20 years	23.0	30.8	9.1	0.0	20.0	33.3	30.8
	21–40 years	8.0	30.8	9.1	0.0	10.0	0.0	30.8
	>40 years	1.0	0.0	4.5	0.0	0.0	0.0	0.0
Type of vegetables sold	Leafy greens	77.0	37.9	40.0	36.4	36.6	63.2	37.9
	Fruity vegetables	60.0	24.1	35.6	36.4	26.8	36.8	24.1
	Root vegetable	33.0	27.6	20.0	27.3	24.4	0.0	27.6
	Edible plant stem	10.0	10.3	4.4	0.0	12.2	0.0	10.3
Food safety training	Yes	54.0	69.2	45.5	80.0	55.0	66.7	69.2
	No	46.0	30.8	54.5	20.0	45.0	33.3	30.8

### 3.2 Compliance with good agricultural and hygienic practices: gap analysis and statistics

#### 3.2.1 Survey analysis farmers

To assess compliance with the good agricultural practices as prescribed by the ‘five keys of growing safer fruits and vegetables’ [7], percentages of complying farmers were calculated in the gap analysis. The results for the farmers are displayed in Table 4, including the assigned color codes. The most dominant gaps for these farmers are: the way of hand washing; document manure application schedule; wash and dry harvest tools; keep containers off the ground before, during and after harvesting; store vegetables in boxes, containers and properly covered. These can be assigned as ‘managerial’ gaps, as they demand a different way of working but do not demand a technological or infrastructural change. Other identified interventions require more technical or technological interventions [32]: protect fields from animal fecal contamination, irrigation water source, clean irrigation systems, test irrigation water quality, irrigation type: avoid use of overhead sprinklers or intentional flooding for ready to eat produce, water source for washing vegetable produce. In the research of Kirezieva et al. [10], a cluster analysis of outcomes of a diagnostic tool to measure the maturity of a food safety management system (based on good agricultural and hygienic practices) demonstrated that farmers and traders active in the non-EU fresh produce chain (based on data collection of small holders in Brazil, Uganda, Kenya, China, India, Egypt and Serbia) are subjected to a less regulated food system with lower policy development towards preventive approach and are less controlled by food safety authorities and an integrated vertical food chain amongst the different (demanding) food actors. This context resulted in a less mature food safety management system and major identified gaps on the level of the design of control activities such as irrigation water source in combination with irrigation system, hand washing, clean utensils (pre- and post-harvest). These are similar results as found in the current gap analysis. International export supply chains promote capacity building within companies in the chain, to answer the stringent requirements of private brand standards [33], however this pressure is lacking in local supply chain as in the current case.

**Table 4:**
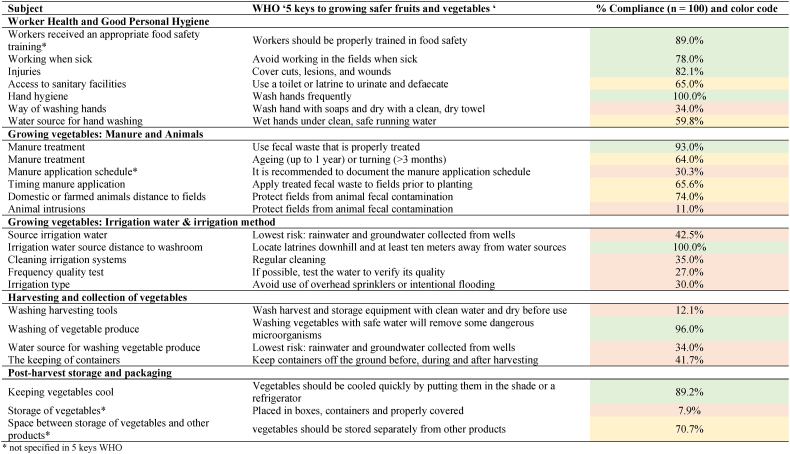
Data on compliance to good agricultural practices for farmers, expressed as %. These good practices are sorted according to the requirements in the WHO's ‘five keys of growing safer fruits and vegetables’ [7] and a gap analysis is conducted (color codes) to retrieve the most important gaps in agricultural practices in our farmers' sample. The color codes: when less than 50 % of farmers executed the practice as prescribed by the ‘5 keys of growing safer fruits and vegetables’ [7], color code red was given and this practice was considered a gap, between 50 % and 75 % of farmers complied: color code yellow, more than 75 % of farmers complied: color code green. *not specified in 5 keys WHO.

Statistical analyses were done to evaluate if various levels of compliance with good agricultural practices could be identified between different farm locations, type of farms (cooperation/family business) and farming activities (vegetables or mixed: livestock and crops). P-values of the Chi-Square Tests of Independence are displayed in Table 5. Most statistically significant differences (significance level 5 %, so p-value <0.05) in compliance to good agricultural practices were found between farms who solely cultivate vegetables (vegetable farmers) and farmers who grow crops and livestock (mixed farmers). Some examples of these differences are: 95.7 % of mixed farmers report to wash harvest containers with clean water (as prescribed by WHO), while 67.1 % of vegetable farmers report the same. Also, 95.7 % of mixed farmers report keeping vegetables cool in the shade (as prescribed by WHO), while 67.1 % of vegetable farmers report the same. 17.4 % of mixed farmers store vegetables in containers that are properly covered (as prescribed by WHO), while 5.2 % of vegetable farmers report the same. These results could be called counterintuitive, as vegetable farmers could be expected to be better acquainted with safe vegetable farming practices compared to mixed farmers. The other characteristics of the farmers were consulted to try to find a possible explanation for this. Adequate education levels are similar between vegetable and mixed farmers, both groups respectively have 50.6 % and 52.2 % of vegetable and mixed farmers who went to high school. 73.9 % of mixed farmers are relatively new to farming with ten or less years of experiences, compared to 35.1 % of vegetable farmers, while simultaneously 82.6 % of mixed farmers have received food safety training (26.3 % less than one year ago), compared to 90.9 % of vegetable farmers (38.6 % less than one year ago). This excludes training and recentness of the training, education levels, and experiences as plausible causes. Lastly, 30.4 % of mixed farmers are part of a farming cooperation, compared to 40.0 % of vegetable farmers. From the above, as no link to other characteristics could be found, it could be speculated that the higher levels of compliance amongst the mixed farmers are due to those mixed farming activities. These farmers might be aware of the risk of cross contamination between animal and vegetable farming, increasing their motivation and therefore compliance levels. It could also be that they are more aware of the importance of food safe practices in general due to their experience in the higher risk context of animal farming. Further research would be needed to confirm this. On the other hand, 47.8 % of mixed farmers treat manure as described in the WHO 5 keys, while this is the case for 67.5 % of the vegetable farmers. Furthermore, none of the mixed farmers report to not use overhead sprinklers or flooding for irrigation (as prescribed by WHO), while this is used by 37.7 % of vegetables farmers.

**Table 5:** P-values of the Chi-Square Tests of Independence to evaluate, for the farmers, if different levels of compliance with good agricultural practices could be identified between different farm locations, type of farms (cooperation/family business) and farming activities (vegetables or mixed: livestock and crops). For the sellers, it was evaluated if different levels of compliance with good hygienic practices could be identified between different groups of sellers based on educational background, experience, and food safety training. When the p-value is indicated in bold this means a significant difference between groups, e.g. the type of farm significantly influences how often food safety trainings are done (p-value <0.001).

Good agricultural practice (farmers)	Location	Type of Farm	Farming activities
How often are food safety trainings done?	**0.009**	**<0.001**	**0.008**
How do workers deal with cuts and other injuries when they occur on farm?	**0.041**	0.080	0.746
What source of water do you use for washing hands?	0.707	0.091	0.762
How is manure treated on your farm?	**<0.001**	**<0.001**	**0.004**
When is the application of manure to vegetable growing fields done?	0.174	0.200	0.235
What is the source of irrigation water on the farm?	**<0.001**	**<0.001**	0.787
How often do you clean your irrigation systems?	0.057	0.661	0.737
How often do you test for the quality of your irrigation water?	0.355	0.291	**0.012**
Which type of irrigation practice is applied?	0.306	0.170	**0.012**
How are vegetable harvesting containers and tools (e.g. knives) washed?	**0.001**	0.077	**0.014**
What is the source of water for washing vegetable produce?	**<0.001**	**0.002**	0.558
How are harvesting containers kept?	0.342	0.097	0.672
How are harvested vegetables kept cool?	0.066	0.055	**<0.001**
How are stored vegetables kept from contamination?	0.152	0.063	**0.007**
**Good hygienic practice (sellers)**	**Educational background**	**Selling experience**	**Food safety training (yes/no)**
How do you wash your hands?	0.881	0.966	**<0.001**
Do you wash vegetables before selling them?	0.647	0.495	0.547
How do you normally store your vegetables before selling them?	0.801	0.391	0.263
What do you do when you are physically unwell?	**<0.001**	**<0.001**	0.724

In the comparison between cooperation farms and family business, some differences in practices could also be noticed. To start, 100 % of cooperation farms report to use a water from a borehole/well or municipal water for irrigation (as prescribed by WHO), while 9.5 % of family business farmers report the same. Secondly, the same trend is logically observed for the water source for washing vegetables: 70.2 % of cooperation farmers report to wash vegetables with water from a borehole/well or municipal water (as prescribed by WHO), compared to 29.5 % of family business farmers. However, concerning the proper treatment of manure (WHO prescription: stacked in a pile and left for a long period of time (up to one year) with no further additions/turning the manure pile for last at least 3 months), 59.5 % of cooperation farmers report to comply while 65.1 % of family business farmers report to comply.

Lastly, a comparison of compliance was also made between the different farms (location) in general. The most interesting results here are the difference in water sources used for various applications. In the farm ‘Hòa Hiệp’, 57.7 % of farmers report to wash containers with any available water and 38.4 % say they use clean water. In all four other farms, more than 80 % reveal to use clean water. In this same farm ‘Hòa Hiệp’, no farmers used water from a borehole/well or municipal water (as prescribed by WHO) as irrigation source, while in the farm ‘Hoa Phong’ this is 100 %.

#### 3.2.2 Survey analysis sellers

To assess sellers' compliance with the good practices as prescribed by the ‘five keys of growing safer fruits and vegetables’ [7], percentages of complying sellers were calculated in the gap analysis. The gap analysis for the sellers in the traditional markets is displayed in Table 6, including the assigned color codes. The most dominant gaps (<50 % compliance) are the way of hand washing (45.0 % of sellers wash their hands with water and soap and dry them with towel), and keeping containers off the ground before, during and after harvesting (18.3 % of sellers reports to keep vegetables of the floor by putting them on table tops or benches before selling them, and 0.0 % reports doing this when displaying vegetables when selling). Storing vegetables off the ground is a reoccurring issue in vegetable selling hygiene practices research, e.g. Abass et al. [34] found that vegetable sellers in Ghana generally stored and displayed their produce in the open, on the ground. Two other gaps were revealed (50–75 % compliance): 54.0 % of sellers report having received any food safety training (compared to 89.0 % of farmers), and 66,0 % of sellers report to wash vegetables before selling (compared to 96.0 % of farmers). These can also be identified as ‘managerial’ gaps [32].

**Table 6:**
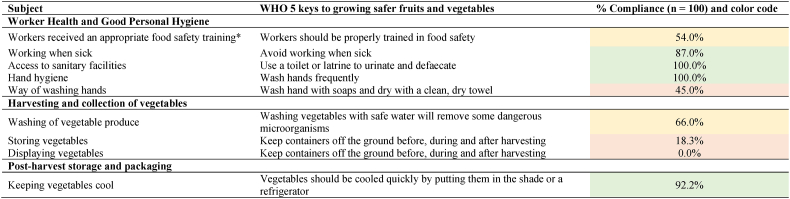
Data on compliance to good hygienic practices for the sellers are expressed in %. Further, these practices are grouped according to the WHO's ‘5 keys of growing safer fruits and vegetables ‘[7] and a gap analysis is conducted (color code). The color codes: when less than 50 % of sellers executed the practice as prescribed by the ‘5 keys of growing safer fruits and vegetables’ [7], color code red was given and this practices was considered as a gap, between 50 % and 75 % of sellers complied: color code yellow, more than 75 % of sellers complied: color code green.

Statistical analyses to evaluate if different levels of compliance with good agricultural practices could be identified between different groups of sellers based on educational background, selling experience and food safety training was conducted and p-values of the Chi-Square Tests of Independence are displayed in Table 5.

The first statistically significant result was found based on food safety training. Hands should be washed with clean water and soap, and dried with a clean towel [7]. 29.6 % of sellers who indicate they have received a food safety training report to wash their hands with clean water and soap, and dried with a clean towel, compared to 63.0 % of sellers who report they did not receive a food safety training. So, contrasting what would be expected, sellers who did not receive any food safety training have higher compliance rates. The second statistically significant result was based on educational background. Sellers had educational background going from no education (n = 2), to elementary education (n = 31), high school (n = 65) and higher education (n = 2), with respectively 100.0 %, 83.9 %, 92.3 % and 50.0 % of sellers indicating that when sick, they close their vending stall until they feel better (as including in [7]). Of course these statistics are influenced by the fact that sellers with no education and higher education represent low percentages of the sample. The last statistically significant result was found based on selling experience. Sellers had <5 years, 5–10 years, 11–20 years, 21–40 years, and more than 40 years of experience. 77.3 %, 93.5 %, 100 %, 75.0 % and none respectively indicate that when sick, they close their vending stall until they feel better (as advised by [7]). Also here, these statistics are influenced by the distribution of the sellers across categories, as one seller has more than 40 years' experience and eight out of 100 sellers have 21–40 years’ experience (Table 3). In this study, a convenience sample of one hundred sellers present in the traditional markets is included. It is not a full representation of all sellers, although we can deduce that the majority of the sellers have no higher education, are not in the oldest age group and are female.

### 3.3 Microbiological analysis (farms and sellers)

Table 7 shows the microbiological results of the samples from farms (F1, 2 and 3) and sellers (MK1, MK2). For farmers and sellers, a total of 36 fresh produce samples (ready-to-eat products), including mustard greens, cucumber, lettuce, and crown daisy), were analyzed for *E. coli* (12 positives in 25 g, range 3.8–5.2 log_10_ CFU/g), *Salmonella* spp. (2 positives in 25 g), *L. monocytogenes* (10 positives in 25 g, range 4.3–6.2 log_10_ CFU/g). Samples of hands showed high contamination with *E. coli* (4 out of five samples were contaminated, range 2.5 to over 5 log_10_ CFU/25 cm^2^), and also the irrigation water was highly contaminated with *E. coli*, demonstrating fecal contamination (2 out of 3 samples, range 4.2 to over 5 log_10_ CFU/100 ml).

**Table 7:** Microbiological results from the farms (F1, F2, F3) and the sellers (MK1, MK2), before the intervention, with the bold indicated values above the applied references according to Table 1.

Farm	Type of sample	Number of samples	Detail	*E. coli* (log_10_ CFU/g or 100 ml or 25 cm^2^)	*Salmonella* spp. (presence or absence in 25 g or 100 ml) (number of positive samples/total samples)	*Listeria monocytogenes* (log_10_ CFU/g or ml or cm^2^)
F1	Produce	3	Mustard greens	<1	0/3	<1
		2	Cucumber	<1	0/2	<1
		3	Lettuce	<1	0/3	<1
		3	Crown daisy	<1	0/3	<1
	Hand	1	Hand	**3.7**^a^	NA	NA
	Irrigation water	1	Irrigation water	<1	0/1	NA
F2	Produce	2	Mustard greens	<1	1/2	<1
		2	Cucumber	<1	0/2	<1
		2	Lettuce	**>5**^a^	0/2	<1
		2	Crown daisy	<1	0/2	**>6**^a^
	Hand	1	Hand	**4.7**	NA	NA
	Irrigation water	1	Irrigation water	**>5**	0/1	NA
F3	Produce	1	Mustard greens	**4.8**	0/1	<1
		1	Cucumber	<1	0/2	**4.3**
		1	Lettuce	**4.4**	0/2	**5.2**
		2	Crown daisy	**5.2**^a^	**1/2**	**4.8**^a^
	Hand	1	Hand	**2.5**	NA	NA
	Irrigation water	1	Irrigation water	**4.2**	0/1	NA
MK1	Produce	2	Mustard greens	**3.8**^b^	0/2	<1
		2	Cucumber	<1	0/2	**4.8**^a^
		2	Lettuce	<1	0/2	**6.2**^a^
	Hand	1	Hand	<0.7	NA	NA
	Washed water	1	Washed water	<1	0/1	NA
MK2	Produce	2	Mustard greens	**4.1** (±1.2)	0/2	<1
		2	Cucumber	<1	0/2	<1
		2	Lettuce	**3.8**^a^	0/2	<1
	Hand	1	Hand	**> 5**	*NA*	NA
	Washed water	1	Washed water	<1	0/1	NA

NA: not analyzed.

a The result of one sample was below the detection limit (<1 log_10_).

b The result of one sample was over 5 log_10_.

Table 7 indicates that samples of fresh vegetables in farms 2 and 3 were contaminated with microorganisms, while samples of vegetables in farm 1 were not. Farm 1 is located in area F1 using irrigation water either from a borehole/well or municipal water, known to be less fecal contaminated [7]. While all of farmers in F2 area (farm 2) used surface water and 33.3 % of farmers in F3 (farm 3) used municipal water, the others made use of surface water. Regarding the water source, farm 2 and farm 3 used free available surface water from ponds and lakes showing an elevated level of fecal contamination, exceeding the safe level compared with those specified in Table 1. It can be seen that the source of irrigation water is one of the main causes of microbial contamination in vegetables.

Moreover, all samples taken from farmers' hands and 1 out of 2 samples from sellers’ hands were contaminated with *E. coli*. These microbiological analysis results are consistent with the findings of the survey on the awareness level of farmers following the 5 WHO keys, 34.0 % of farmers and 45.0 % of sellers adhere to hand washing with soap and drying time, and less than 50.0 % of farmers use safe water to wash their hands at F1, 2, 3. Thus, it can be seen that the water used to wash hands and how to wash hands is a high-risk factor for *E. coli* infection from farmers' hands to grown vegetables.

### 3.4 Managerial and technological interventions, post-assessment and lessons learned

**Fig. 2:**
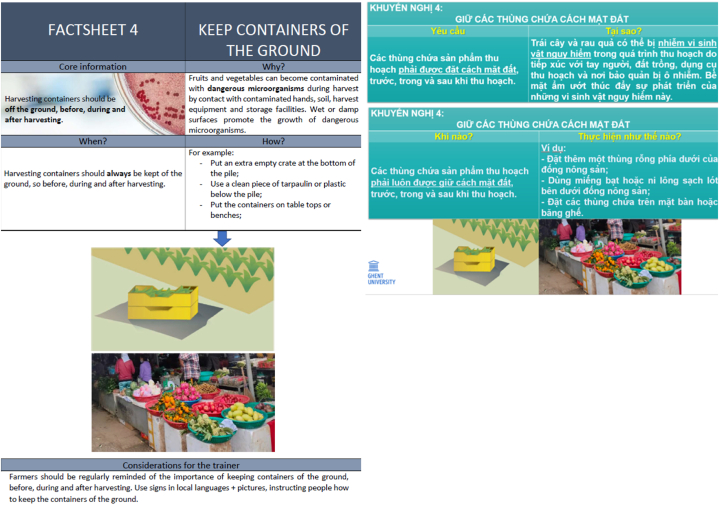
Example of designed factsheet four ‘keeping containers off the ground, before, after and during harvesting’ based on five keys of WHO [7] (left) and tailored, based on the science-based gap analysis to the situation of small holders and sellers in traditional food markets (right).

After the science-based gap analysis, ten factsheets were designed and shared with all sellers and farmers (n = 200) based on the 5 keys of WHO [7] for each of the identified gaps for the farmers and sellers (i.e. washing hands, documented manure application, irrigation water source and irrigation type etc.). These were tailored to the Vietnamese context by a translation to Vietnamese, but also adaptation towards the typical regional context situation e.g. examples of suitable available water sources, intrusion of local (wild) animals, typical produce cultivated in the area), to enhance the understandability and recognition for the farmers and sellers. Adapting interventions to the local context and subjects' needs has been proven essential in multiple studies (e.g. [35]). Fig. 2 shows an example of the gap of ‘keeping containers of the ground, before, after and during harvesting’, before and after the tailoring to the Vietnamese situation and context.

Furthermore, to capture the main messages from the more elaborated factsheets in a short and profound manner, a roadmap was designed as a risk communication tool. Both the factsheets and the roadmap were distributed to all two hundred farmers and sellers. A tailored training using these tools was then done with a limited set of two farmers and two sellers, in order to transfer knowledge and create awareness on how to reduce the potential microbiological contamination of the fresh produce, as a managerial intervention. Therefore, a face-to-face training and coaching by researchers from the Da Nang university were conducted in April–May 2022. Additionally, as a technological intervention at farm level, tap water was applied as irrigation water instead of the uncontrolled surface water for the production of leafy greens in dedicated fields in May–June 2022. After elaborating the intervention (both managerial and technological), a post-assessment and sampling round was done at the famers and sellers to evaluate the potential impact of these interventions (June 2022). Similar sampling and microbiological analysis as during the gap analysis was performed (Table 1). Microbiological outcomes at farm level and seller level, as post-assessment of the interventions are represented in Table 8.

**Table 8:** Microbiological results from the farmers and sellers, after the intervention, in bold indicated values are above the applied reference values (Table 1).

Market/farm	Type of sample	Number of samples	Detail	*E. coli* (log_10_ CFU/g or 100 ml or 25 cm^2^)	*Salmonella* spp. (presence or absence in 25 g or 100 ml) (number of positive samples/total samples	*Listeria monocytogenes* (log_10_ CFU/g or ml or cm^2^)
F1	Produce	3	Mustard greens	<1	0/3	<1
		3	Crown daisy	*1*^a^	0/3	<1
	Hand	1	Hand	<0.7	NA	NA
	Irrigation water^b^	1	Irrigation water^b^	<1	0/1	NA
F3	Produce	3	Mustard greens	<1	0/3	<1
		3	Crown daisy	<1	0/3	<1
	Hand	1	Hand	<0.7	NA	NA
	Irrigation water^b^	1	Irrigation water^b^	<1	0/1	NA
MK1	Produce	2	Mustard greens	**3.4**^a^	0/2	<1
		2	Cucumber	<1	0/2	<1
		2	Lettuce	**>5**^a^	0/2	<1
	Hand	1	Hand	<0.7	NA	NA
	Wash water^b^	1	Wash water	<1	0/1	NA
MK2	Produce	2	Mustard greens	<1	0/2	<1
		2	Cucumber	<1	*−*0/2	<1
		2	Lettuce	1.6^a^	0/2	<1
	Hand	1	Hand	<0.7	NA	NA
	Wash water^b^	1	Wash water	<1	0/1	NA

NA: not analyzed.

a The result of one sample was below the detection limit (<1 log_10_).

b During the intervention the applied water was municipal water, so potable drinking water.

Although the sample size from the farmers and sellers involved in the supervised training sessions and technological intervention is limited (n = 4), it is clear that both the managerial interventions (training and creating awareness on food hygiene and microbiological safety) and the technological invention (application of tap water instead of fecal contaminated surface water) has a major impact on the microbiological contamination of the produce and the generic hygiene of the workers. At the farm level, one sample contained an acceptable contamination of 10 CFU/g E*. coli*. For the sellers, two samples were still higher contaminated for *E. coli*. However, the use of tap water as agricultural water is debatable (in terms of sustainability and need for such a high quality of water for the application). This intervention was applied as a case study to demonstrate the impact of the water contamination on the produce contamination. In practice, it would not be feasible to request farmers to apply tap water for irrigation in the long run. International guidance documents recommend use of ‘clean’ water with max. 100 CFU/100 ml E*. coli* for overhead irrigation of ready to use fresh produce (e.g. Commission [36], Commission Notice, 2017). Therefore, the use of well water or collected rainwater can be further explored in the Da Nang region instead of the current use of uncontrolled surface water, to enhance the hygiene of the fresh produce on the one hand and to reach sustainable use of high quality water for agricultural practices on the other hand [37]. In previous research in Europe, agricultural water was also identified as a major risk factor in microbiological contamination of fresh produce and clear links could be made between open, unprotected water sources and low quality of the water [3]. The approach on implementing a food safety management system based good agricultural and hygienic practices has been widely demonstrated to be effective in other emerging countries (e.g. [4] (basil production of South Africa) [5]; (lettuce production in South Brazil) [6]; (lettuce production of South Africa)).

According to Unnevehr [9] critical research in food safety governance needs to focus on two broad areas. First, risk analysis is needed at the regional or country level in order to fully understand risk exposure, and to determine the most important public health targets for policy intervention. Secondly, research is needed to find the best models for intervention that can leverage market incentives. Refocusing research on these needs, rather than on export supply chains, can support improved food safety, and ultimately improved food security [9]. Governing food safety in a food system is a multidimensional responsibility from farm to plate but also in terms of macro, meso and micro actors in the food system [33]; [9].

Based on these principles and the outcomes of the presented research at macro level (governmental and food safety authority, food policy level) two major suggestions can be made for the particular case of fresh produce chain in the Da Nang region. Firstly, on macro level, food safety governance is (still) fragmented since farmers are not considered as food business but depend on the Ministry of Rural Development. The Da Nang food safety management authority cannot control compliance to good agricultural and hygienic practices or sample at the farms. This situation has an impact on the farm-to-plate approach in food safety management because different governmental organisations or ministries each have a part of the food system under their responsibility. The fragmented situation may lead to other priorities or miscommunication amongst each other [38]. Secondly, the source of irrigation water in the region is a concern as mostly uncontrolled and highly variable surface water is applied. It was clear that produce had fecal contamination during the microbiological assessment (Table 7). According to international guidelines, using less contaminated water for irrigation of ready to eat leafy greens in an overhead sprinkler system is essential [7], Commission Notice, 2017. This is an issue that a single farmer cannot solve, as he/she needs to use the water available in their cultivation area. The policy-makers in the region of Da Nang can intervene to seek for suitable water sources for ready to eat and high-risk leafy greens. Managerial interventions on GAP and GHP in combination with the technical intervention (switch to less contaminated water source) with the farmers and the sellers have clearly revealed a much better microbiological hygiene and quality compared to pre-intervention status. Rolling out the acquired knowledge and tools for training and risk communication in the broader area is of utmost importance. The local university in Da Nang, as meso actor in the food system, can help to elaborate further in the knowledge transfer and capacity building in the region by lecturing food technology students, or provide extension work towards farmers and sellers. A broader communication campaign can be set up, making use of the factsheets and the roadmap. Finally, at micro level, the small-scale farmers and sellers in the traditional markets can be encouraged in implementing the GAPs and GHPs through the inspections and guidance conducted by the food safety authority or farmer organisations, without the need of going into a certification system or other market driven reward system (such as Viet GAP). It is not the objective to shift the traditional markets, which are sustainable and locally driven, towards partnerships with the private sector to support supply chain coordination as is the case in many high value markets. The literature to date has focused primarily on export markets, but much more could be done to evaluate the potential of these efforts in domestic markets in order to identify best practices [9].

## 4 Conclusions and future perspectives

In this research, major gaps in good agricultural and hygienic practices in small-scale farming of ready to eat leafy greens in the Da Nang region in Viet Nam were identified at farm and seller level. For the farms, the managerial gaps of washing hands, documenting the manure application schedule, washing, and drying harvest tools, keeping containers off the ground, storing vegetables in boxes, and properly covering containers were identified. Gaps were also identified that were linked to technical (infrastructural) interventions (e.g. irrigation water source or clean irrigation systems). A single farmer cannot solve this on his own and therefore, governmental support and ambition are needed, e.g. in finding clean water irrigation sources (e.g. collected rainwater or well water). Based on the gaps risk communication tools were developed and shared (managerial intervention). Furthermore, two sellers and two farmers were selected, based on their availability, to join training (managerial intervention) and technical interventions (safer source of irrigation water). Interventions clearly demonstrated improved safety of the products.

For obtaining a long term increase in food safety levels in the studied fresh produce value chain, a collaboration in food safety management is needed between the macro level, to develop adequate policies and facilitate proper sources of clean water (i.e. the food safety authority and the ministry of agriculture), the meso level (i.e. the university, food technology department) that can collect objective information in measuring the status of hygienic practices and can develop training material for the farmers and sellers and, finally, the micro level where knowledge and risk awareness can be created amongst farmers and sellers. This way, an integrated farm to plate governance could be achieved, guaranteeing the safety of the produce. This research demonstrated a risk management strategy in an agri-food chain consisting of smallholders with limited vertical integration, and hence limited control over the supply chain, in a particular food chain in a particular region. The presented mode of working can be transferred to other food chains and regions to enhance the traditional food chains in governing food hygiene and safety.

However, it is important to acknowledge certain limitations of this study. Firstly, the research was conducted at specific areas of Da Nang in Viet Nam. This may limit the applicability of the findings to other areas. Future studies should incorporate a more diverse sample from various regions to gain a more comprehensive understanding of the factors. Moreover, due to Covid and logistic restrictions at the period of research, the sample size from the farmers and sellers involved in the supervised training sessions and technological intervention was limited. Future research may consider including more farmers and sellers in the supervised intervention to enhance and validate the findings. Future research can also focus on longitudinal studies to track changes over time (our interventions were running for several weeks) or interventions targeting specific gaps (e.g. technological intervention of improved microbiological quality of the available irrigation water). In summary, this study contributed to identifying the main risks and hotspots in Da Nang's fresh vegetable value chain based on an evidence-based analysis of food safety gaps and developing tailored roadmaps for improvement for different actors in the fresh vegetable supply chain. The findings can serve as a model for the development of science-based risk management in food safety in other food chains and/or in other regions of Viet Nam, where limited data are available on microbiological status and food safety management systems.

## Funding

This research was conducted under the funding of 10.13039/501100022083VLIR-UOS South Initiative project ‘Towards a risk-based food safety management system in Da Nang, Viet Nam’ (SI-2020-01-32) (2020–2022).

## Informed consent statement

Farmers were approached through Farmers' Associations of the communes/wards and sellers were approached through the Management Board of Traditional Markets. They participated in the survey on a voluntary basis as their names were filled in the form and signed the survey list and all data were ammonized during the data processing and reporting. Da Nang Food Safety Management Authority agreed to participate in the research and signed a cooperation agreement with The University of Da Nang - University of Science and Technology in the fields of training, scientific research, academic exchange.

## Data availability statement

**Data Availability**.

Sharing research data helps other researchers evaluate your findings, build on your work and to increase trust in your article. We encourage all our authors to make as much of their data publicly available as reasonably possible. Please note that your response to the following questions regarding the public data availability and the reasons for potentially not making data available will be available alongside your article upon publication.

Has data associated with your study been deposited into a publicly available repository?

No.

Please select why. Please note that this statement will be available alongside your article upon publication. as follow-up to “**Data Availability**".

Sharing research data helps other researchers evaluate your findings, build on your work and to increase trust in your article. We encourage all our authors to make as much of their data publicly available as reasonably possible. Please note that your response to the following questions regarding the public data availability and the reasons for potentially not making data available will be available alongside your article upon publication.

Has data associated with your study been deposited into a publicly available repository?Data included in article/supp. material/referenced in article.

## CRediT authorship contribution statement

**Thanh Ha Thi Mac:** Writing – review & editing, Writing – original draft, Formal analysis, Data curation. **Thi Dong Phuong Nguyen:** Writing – original draft, Investigation, Formal analysis. **Minh Nhat Dang:** Writing – review & editing, Supervision, Project administration. **Thi To Quyen Ta:** Writing – original draft, Investigation. **Pauline Spagnoli:** Writing – review & editing, Writing – original draft, Formal analysis. **Mieke Uyttendaele:** Writing – review & editing, Supervision, Project administration, Methodology. **Liesbeth Jacxsens:** Writing – review & editing, Writing – original draft, Methodology, Data curation.

## Declaration of competing interest

The authors declare the following financial interests/personal relationships which may be considered as potential competing interests:Minh Nhat Dang reports financial support was provided by 10.13039/501100022083VLIR-UOS. If there are other authors, they declare that they have no known competing financial interests or personal relationships that could have appeared to influence the work reported in this paper.

## Figures and Tables

**Table:** 

VLIR PROJECT (TOWARDS A RISK-BASED FOOD SAFETY MANAGEMENT SYSTEM IN DA NANG, VIETNAM) QUESTIONNAIRE ON OF GOOD AGRICULTURAL PRACTICES (GAP) AND GOOD HYGEINIC PRACTICES (GHP) AT **FARM** LEVEL IN DA NANG, VIETNAM Filled by:______________________________________ Date:_________________________________________ Farmer ID:_____________________________________ SECTION 1: Background Data 1. Age a 15–18 years (adolescents) b 19–29 years (adult group 1) c 30–65 (adult group 2) d >70 years (elderly) 2. Gender a Male b Female 3 Educational background a. None b Elementary school c High school d Higher education 4. Farming experience a <5 years b 5–10 years c 11–20 years d 21–40 years e > 40 years 5. Type of vegetable/produce grown: a Leafy greens- spinach, lettuce, cabbage b. Fruity vegetables- tomatoes, cucumber, eggplant c. Root vegetable- carrot, sweet potato, turnips d. Edible plant stem- celery, asparagus, kohlrabi 6 Any food safety training a Yes b . No 7. If yes, how long ago: a < 1 year b 1–3 years c > 3 years ago 8 Type of farm a. Family business b Cooperation 9. Farming activities: a Solely vegetables/fruits/cereals/crops b. Mixed activities (livestock and crops) c. Mixed activities (crops and aquaculture) SECTION 2: Good Agricultural and Hygienic Practices **A. Worker Health and Good Personal Hygiene** 1. Are workers properly trained in food safety practices a Yes b No 2. How often are these trainings done? a. Once a year b Twice a year c Every two years d Every five years 3. What do you do when you or a worker is physically unwell a. continue to work on farm b. seek absence until physically able to work c. lie around the farm 4. How do workers deal with cuts and other injuries when they occur on farm a. cuts and or abrasions are treated as minor inconveniences and left as is b. wash it under running water and continue working c. clean it and keep covered 5. How far are washroom facilities from your farm a. 50 m (66 steps) b 100 m (131 steps) c 150 m (197 steps) d more than 200 m e. no washrooms available on the farm (where also the workers can make use of) 6. Do you regularly wash your hands a Yes b No 7. If yes, how often do you do so a. up to 2 times a day b 3–5 times a day c. more than 5 times a day d as often as required 8. When do you normally wash your hands a. at start of work b after visiting the toilet c removal of dirt/garbage d before handling farm produce 9. How do you wash your hands a. with water b with water and soap c with water and soap and dry with towel 10. What source of water do you use for washing hands a piped water b dug well/borehole c from storage tanks/containers d river/pond e. other (specify)________ **B. Growing vegetables: Manure and Animals** 11. Is manure that is composted on the farm properly treated? a Yes b No 12. If yes, how is manure treated on your farm? a. Storing manure in pits and covering for a short period of time b. Stacking in a pile and leaving it for more than a year with no further additions c. Stacking in a pile and leaving it for less than a year with regular new additions d. turning the manure pile and periodically exposing to air to accelerate decay 13. Is the manure application schedule documented aYes bNo 14. When is the application of manure to vegetable growing fields done? a prior to planting b during planting c after harvesting 15. How far do you keep domestic or farmed animals from vegetable growing fields a 50 m (66 steps) b 100 m (131 steps) c 150 m (197 steps) d more than 200 m 16. Do you experience intrusions from animals on the fields? aYes bNo 17. If yes, what type of animals? a birds b rats c reptiles d domestic animals 18. Which preventive measures do you take to prevent such intrusions a. buffering zone between forest and field b. place enclosure around the field c. set up mechanisms such as noise alarms and scare crows d. remove trash from around the growing fields to keep animals and birds away **C. Growing vegetables**: **Irrigation water & irrigation method** 19. What is the source of irrigation water on the farm a lake/pond b river c irrigation canal d municipal water e. borehole/well 20 How close is your source of irrigation water to a refuse site/washroom a 50 m (66 steps) b 100 m (131 steps) c 150 m (197 steps) d more than 200 m 21 How do you prevent water source from contamination? a. Place barriers or fences around water source to prevent animals or human intruders b. Cover wells or boreholes and openings of water storage tanks properly c. Washrooms, stored manure, and refuse sites are to be built at least 100 m away from water sources 22. How often do you clean your irrigation systems a Weekly b Monthly c Bi-yearly d Yearly e. Never 23. How often do you test for the quality of your irrigation water a Once every two years b Never c Once every five years d Once a year e Twice a year 24. Which type of irrigation practice is applied? a flood b overhead c irrigation d drip **D. Harvesting and collection of vegetables** 25 How are vegetable harvesting containers and tools (e.g. knives) washed a With any available water b With clean water c With clean water, soap and try towel d Never washed 26. Are vegetable produce washed after harvesting a Yes b No 27 If yes, what is the source of water? a canal (big) b lake/pond c river d irrigation canal (small) e municipal water 28 How are harvesting containers kept a on bare floor b on material on floor c on benches, tables, or pellets **E. Post-harvest storage and packaging** 29. How are harvested vegetables kept cool a in a shade out of the sun b in refrigerators c on bare floor d. in water in baskets 30. How are stored vegetables kept from contamination a. Placed in boxes, containers and are left open b. Left on materials on the floor c. Placed in boxes, containers and properly covered 31. How far are vegetables stored separately from other products? (e.g. fish, meat, eggs, meat) a less than 10 feet b . more than 10 feet c 20 feet or more

**Table:** 

VLIR PROJECT (TOWARDS A RISK-BASED FOOD SAFETY MANAGEMENT SYSTEM IN DA NANG, VIETNAM) – UGENT QUESTIONNAIRE ON GOOD HYGIENINC PRACTICES AT **SELLING POINTS/TRADITIONAL MARKETS** IN DA NANG, VIETNAM Filled by:______________________________________ Date:_________________________________________ Farmer ID:_____________________________________ SECTION 1: Background Data 1.Age a15–18 years (adolescents) b19–29 years (adult group 1) c30–65 (adult group 2) d>70 years (elderly) 2.Gender aMale bFemale 3 Educational background a.None bElementary school cHigh school dHigher education 4.Trading experience (in vegetables) a<5 years b5–10 years c11–20 years d21–40 years e> 40 years 5.Any food safety training aYes b. No 6. Which vegetables do you sell ___________________________________________ SECTION 2: Good Hygienic Practices 1. Do you regularly wash your hands? aYes b. No 2. If yes, how regularly do you do so a up to 2 times a day b 3–5 times a day c more than 5 times a day 3. When do you normally wash your hands a at start of work b after handling money c removal of dirt/garbage d before handling vegetables e after visiting the toilet 4. How do you wash your hands a with just water b with water and soap c with water and soap and drying with towel 5. Do you wash vegetables before selling them aYes b. No 6. If yes, which source of water do you use in washing vegetables a piped water b dug well/borehole c from storage tanks/containers d. other (specify)________ 7. How far are washroom facilities from your stall a 50 m (66 steps) b 100 m (131 steps) c 150 m (197 steps) d more than 200 m 8. How do you normally store your vegetables before selling them a unpacked on the floor b packed on the floor (baskets, boxes) c on table top/benches d others________ 9 Where do you display your vegetables for sale? a table top/benches b basket/bowl c material on ground d bare ground e other(specify)___________ 10. How close are trash and rubbish heaps, open drains and animals allowed near your vending stall a 50 (66 steps) b 100 m (131 steps) c 150 m (197 steps) d more than 200 m 11. What do you do when you are physically unwell a. continue to sell produce b. close vending stall until physically able to sell c assign someone to man the stall 12. How are raw vegetables kept cool a in a shade out of the sun b in refrigerators c on bare floor d in water in baskets

**Appendix 3:**
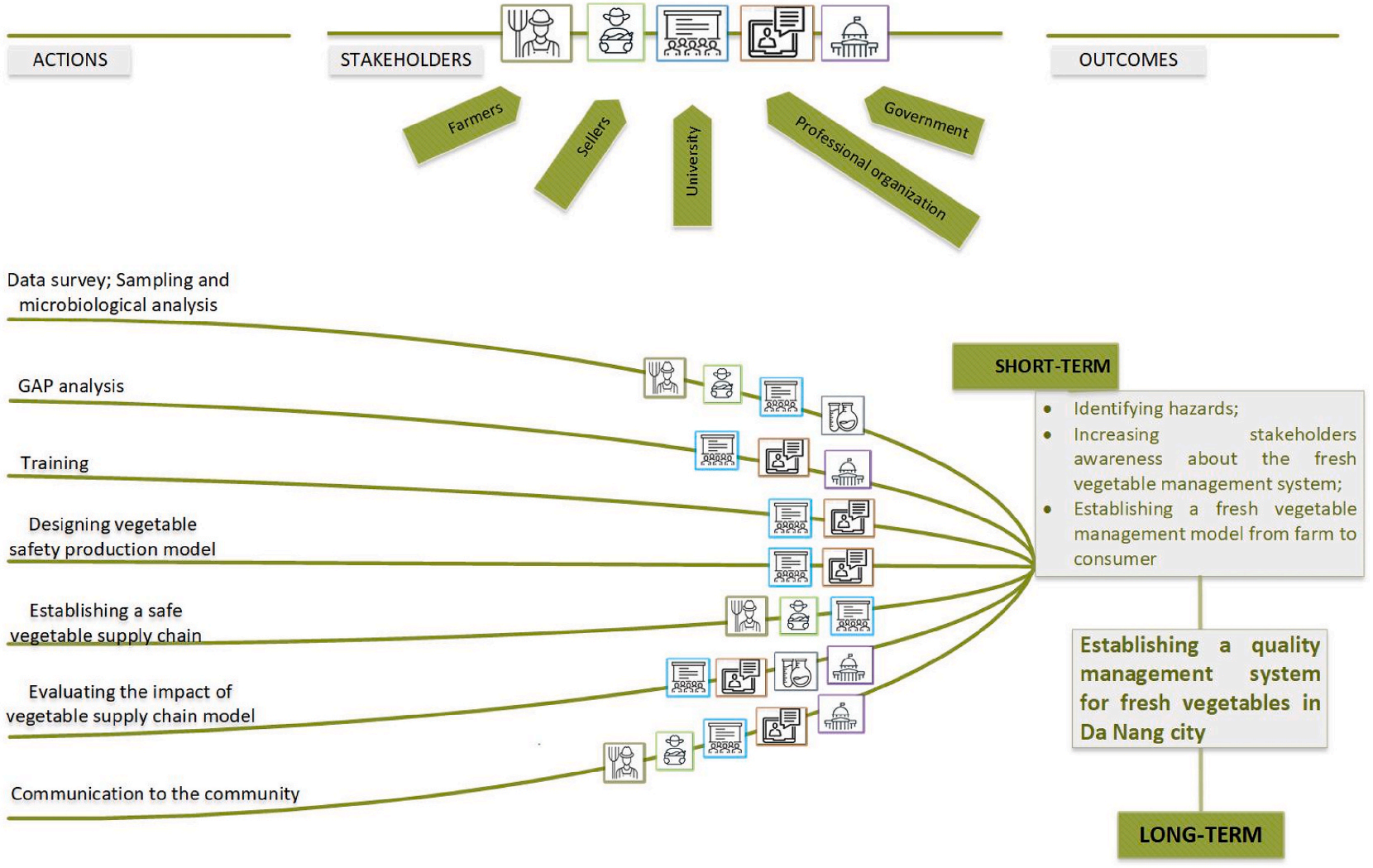
The design of research methodology.
